# Dynamic mapping of network-level LTP in the hippocampus via high-resolution bioelectrical sensing

**DOI:** 10.1063/5.0258985

**Published:** 2025-07-29

**Authors:** Shahrukh Khanzada, Xin Hu, Brett Addison Emery, Władysław Średniawa, Daniel K Wójcik, Gerd Kempermann, Hayder Amin

**Affiliations:** 1 Group of “Biohybrid Neuroelectronics (BIONICS),” German Center for Neurodegenerative Diseases (DZNE), Tatzberg 41, 01307 Dresden, Germany; 2 Laboratory of Neurophysiology of Mind, Centre of Excellence for Neural Plasticity and Brain Disorders (BrainCity), Nencki Institute of Experimental Biology of Polish Academy of Sciences, 3 Pasteur Street, Warsaw 02-093, Poland; 3 Laboratory of Neuroinformatics, Nencki Institute of Experimental Biology of Polish Academy of Sciences, 3 Pasteur Street, Warsaw 02-093, Poland; 4 Group of “Adult Neurogenesis,” German Center for Neurodegenerative Diseases (DZNE), Dresden, Germany; 5 Center for Regenerative Therapies TU Dresden (CRTD), Fetscherstraße 105, 01307 Dresden, Germany; 6 TU Dresden, Faculty of Medicine Carl Gustav Carus, Bergstraße 53, 01069 Dresden, Germany

## Abstract

Understanding the complexity of neural network dynamics demands advanced biosensing technologies capable of capturing large-scale interactions with high spatial and temporal precision. Traditional approaches, such as patch-clamp and field recordings, are inherently limited in resolving network-wide synaptic connections, particularly long-term potentiation (LTP), due to their localized scope and indirect access to hippocampal subfields. To address these challenges, we introduce EvoNES, a CMOS-based high-definition 4096 microelectrode array platform that leverages bidirectional stimulus-responsive biosensing functionality. By coupling precise external electrode stimulation targeting the Schaffer collateral and medial perforant pathways with simultaneous on-chip bioelectrical recordings, EvoNES enables the first real-time quantification of evoked responses and LTP dynamics across the entire hippocampal circuit. This system bridges critical gaps in traditional techniques, providing a mesoscopic-scale view of cell assemblies interplay and delivering unprecedented insights into the distributed mechanisms underlying memory encoding and learning processes. Advanced computational analyses generate variation maps revealing distinct voltage fluctuation patterns and differential sensitivity across hippocampal subregions during synaptic potentiation. Our findings identify four distinct waveform classes within the CA1–CA3 network and three unique evoked firing patterns in the dentate gyrus (DG). Post-tetanic responses show faster induction, expanded activated zones, and the activation of previously silent cell assemblies, indicating significant network restructuring. Applied in aged mice, EvoNES demonstrates age-dependent changes in network LTP, both quantitatively and qualitatively. This high-resolution biosensing platform in a live neural context provides unprecedented insights into hippocampal memory formation and offers a powerful tool for investigating neural plasticity and network interactions in both health and disease states.

## I. INTRODUCTION

Memory, a pivotal cognitive function, is more than just a repository of our acquired information. It is a dynamic neurobiological construct that intertwines with our perceptions, behaviors, and anticipations of the future.^1^ Learning and memory involve a cascade of molecular, cellular, and neural circuit events that encode, store, consolidate, and retrieve experiences, shaping our cognitive and behavioral repertoire.^2^ Like a kaleidoscope creating patterns through mirror rotations and glass arrangements, memories are believed to be represented by coordinated activity patterns through the interaction of cell assemblies across distributed brain networks during specific experiences and behaviors.^3–5^ Despite extensive research, the emergence of memories through large-scale neuronal interactions remains elusive. Understanding the computational dynamics of memory mechanisms can illuminate cognitive capacities and facilitate interventions for memory disorders. While multiple brain regions contribute to various aspects of learning and memory, the hippocampus is central to spatial and episodic contexts, with its unique architecture and specialized cell types enabling the encoding of experiences into long-lasting memory traces.^6–8^ Within the hippocampal circuit, information flows through interconnected regions—the dentate gyrus (DG), CA3, CA2, and CA1—each contributing uniquely to the processing and storage of memories.^6,9,10^ The transmission is facilitated by pathways like the perforant path, mossy fiber, and Schaffer collaterals, modulated by synaptic plasticity.^11^ Activity-dependent synaptic plasticity, particularly long-term potentiation (LTP), has been identified as a cellular substrate for learning and memory, involving intricate presynaptic and postsynaptic modifications.^12^

LTP is categorized by persistent enhancement in synaptic transmission following high-frequency stimulation, while long-term depression (LTD) is characterized by a durable decrease in synaptic strength. LTP and LTD modulate synaptic connections within cell assemblies, enabling efficient signal transmission essential for memory encoding, consolidation, and recall.^13^ This modulation, orchestrated by synaptic plasticity, allows for forming and stabilizing specific sequential firing patterns of particular cell assemblies, embodying the physical substrate of memory engrams.^14,15^

LTP and synaptic plasticity in the hippocampus have been widely investigated using sharp electrode recordings, patch-clamp techniques, extracellular field measurements, and conventional microelectrode array (MEA) biosensors.^16–18^ This has been significantly enriched by the employment of brain slice models, providing a controllable environment to dissect the cellular and network mechanisms underlying these phenomena.^19^ Despite the invaluable insights provided by these methodologies, several limitations have emerged. These include the lack of spatial resolution to accurately map synaptic activity-dependent changes and significant inter-experimental variability that hinder the reproducibility of findings and mask subtle alterations in synaptic plasticity and LTP under different experimental conditions. These limitations have underscored the need for novel large-scale multi-site biosensing techniques that will not only enhance our understanding of the cellular and network mechanisms underlying LTP but also provide a comprehensive spatiotemporal dynamic view of cell assemblies computations in different hippocampal subfields underlying learning and memory processes.^20–22^

Advanced electrophysiological recording techniques employing high-density CMOS-MEAs epitomize the forefront of biosensing innovations, dramatically enhancing our understanding of multimodal neural dynamics. These platforms enable precise, simultaneous recording from thousands of sites, offering an unprecedented spatiotemporal resolution that captures a comprehensive view of neural interactions. This capability is pivotal for bioelectrical sensors aimed at decoding complex neural functions and interactions.^22–26^ In this study, we introduce EvoNES, a cutting-edge, high-density biosensing platform designed for long-term, label-free monitoring of network-level LTP and extracellular evoked synaptic potentials within the hippocampal circuit. This platform integrates state-of-the-art CMOS-MEA technology, reinforcing its role as a critical tool in biosensing applications that require detailed neural mapping and analysis. By integrating large-scale neural recordings from 4096 microelectrodes, advanced computational tools, and insights into neural dynamics, this study aims to map the reconfiguration of large-scale cell assemblies underlying learning processes and memory encoding through studying network-wide LTP and synaptic plasticity.

Our method facilitates bidirectional stimulus-responsive biosensing functionality from two hippocampal canonical pathways—medial perforant path (mPP) to the DG and Schaffer collaterals (SCs) from CA3 to CA1. We use automated spatial and temporal waveform-based classification to align cellular layers of network-induced synaptic responses with their anatomical counterparts within hippocampal subfields. These field-excitatory-postsynaptic potentials (fEPSPs) and the repertoire of induced synaptic responses are identified simultaneously from a firing group of cell assemblies encoded in DG, CA3, and CA1. Our network-wide recordings also enable detailed computations of neuronal transmembrane current sources and sink generators using the kernel current source density (kCSD) method.^27^ Finally, we evaluate network-wide evoked representations in aging hippocampal circuits to reveal how deactivated cell assemblies contribute to functional remodeling.^28^ Our findings indicate that aging diminishes synaptic plasticity by reducing specific firing patterns in targeted hippocampal layers, leading to significant alterations in large-scale memory-coding networks. This study provides insights into how age-related changes in synaptic efficacy and connectivity affect the overall organization and functionality of hippocampal circuits.

This is the first report on label-free, large-scale mapping of functional synaptic activity-dependent changes and network-level LTP induction simultaneously modulated by electrical stimulation in the hippocampus. While primarily aimed at understanding learning and memory with unprecedented biosensing spatiotemporal resolution, our research has broader implications. The platform paves the way for advancements in neuroscience, technology, artificial intelligence, and adaptive learning systems, potentially revolutionizing lifelong learning machines, neuromorphic computing, and brain-inspired machine learning algorithms.^29,30^

## II. RESULTS

### A. Implementation of the EvoNES platform

**FIG. 1.**
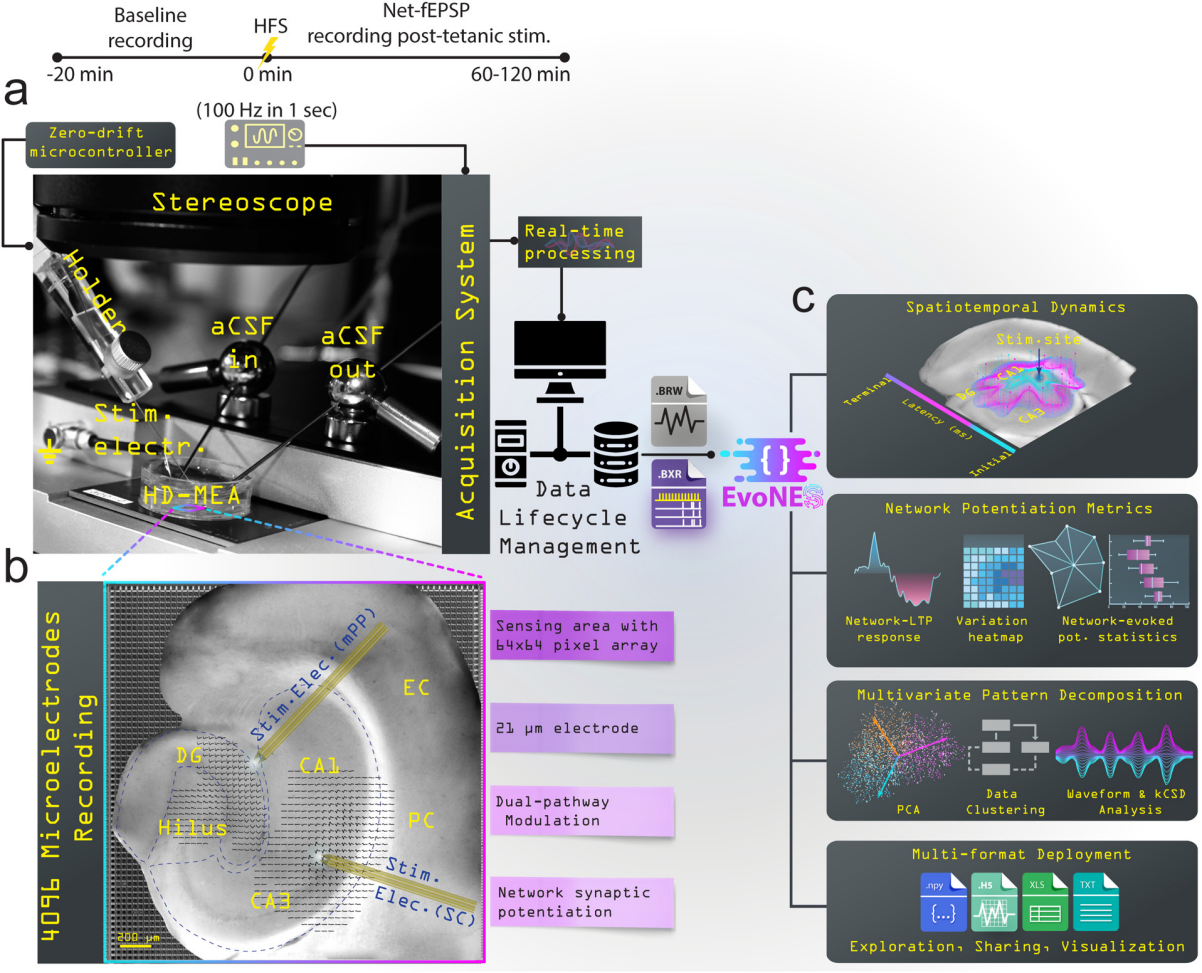
Overview of the EvoNES platform with bidirectional stimulus-responsive biosensing functionality for network-level synaptic and LTP measurement and analysis. (a) The platform facilitates advanced synaptic transmission and network-level LTP investigation across the hippocampal network using HD-CMOS-MEA with 4096 microelectrodes. The setup features a zero-drift triple-axis micromanipulator system for precise stimulating electrode positioning and a custom-designed stereomicroscope for accurate alignment with hippocampal tissue. The experimental workflow includes a timeline from baseline measurement to tetanic-evoked potentiation achieved with a single high-frequency stimulation (HFS) at 100 Hz. (b) The system can stimulate the Schaffer collaterals (SCs) and medial perforant pathway (mPP) to measure simultaneous network-wide evoked activity, capturing distinct EPSP and PS signatures across CA1–CA3 and DG layers using bipolar stimulating electrodes. (c) Overview of the integrated Python-based analytical pipeline implemented to process and visualize high-dimensional neural data. The pipeline extracts spatiotemporal features, computes network potentiation metrics, generates multi-layer waveform representations, and applies clustering and multivariate pattern analysis to characterize LTP dynamics across hippocampal layers. This platform offers flexible, multi-format deployment options for in-depth exploration, data sharing, and visualization.

To comprehensively investigate synaptic transmission and LTP across the intricately hippocampal network, we developed the **Evo**ked **N**etwork **E**lectrophysiology **S**ensor (EvoNES), which combines advanced experimental-computational features (Fig. 1). Central to EvoNES is a high-density planar CMOS-based microelectrode array (HD-CMOS-MEA) with 4096 microelectrodes,^24,25^ offering an advanced biosensing solution for simultaneous, large-scale recording and stimulation. This platform transcends traditional electrophysiological limitations by enabling real-time mapping of functional neural dynamics with unprecedented spatial and temporal resolution. Designed as a multifunctional biosensing tool, EvoNES integrates precision stimulation to the neural tissue through external platinum bipolar electrodes connected through a specialized input–output module to minimize stimulation artifacts. A zero-drift triple-axis micromanipulator ensures precise and stable electrode positioning, while a custom-designed stereomicroscope aligns hippocampal tissue with the CMOS-MEA, optimizing recorded signal fidelity and accuracy [Fig. 1(a)]. The system captures multi-layered synaptic responses across CA1–CA3 and DG networks, bridging critical gaps in neural monitoring technologies and enabling high-throughput, label-free exploration of neuronal plasticity. The dual pathway stimulation through the Schaffer collaterals (SCs) and medial perforant pathways (mPP) can be induced using two external electrodes or targeted stimulation within a specific CA1–CA3 or DG network [Fig. 1(b)]. The platform's further features an integrated Python-based computational framework to process and analyze the extensive multidimensional datasets, extracting spatiotemporal features such as network potentiation maps, multi-layer waveforms, and clustering algorithms. It includes tools for frequency–time dynamics, kernel current-source density (kCSD) analysis, and various statistical metrics, providing a comprehensive understanding of real-time network dynamics rooted in synaptic plasticity [Fig. 1(c)]. The method enables a nuanced exploration of network synaptic dynamics, setting the stage for a detailed investigation into how these dynamics underpin network-wide LTP and its role in memory encoding, as we will explore in Sec. II B.

### B. Characterization of sequential encoding patterns of network-wide LTP

**FIG. 2.**
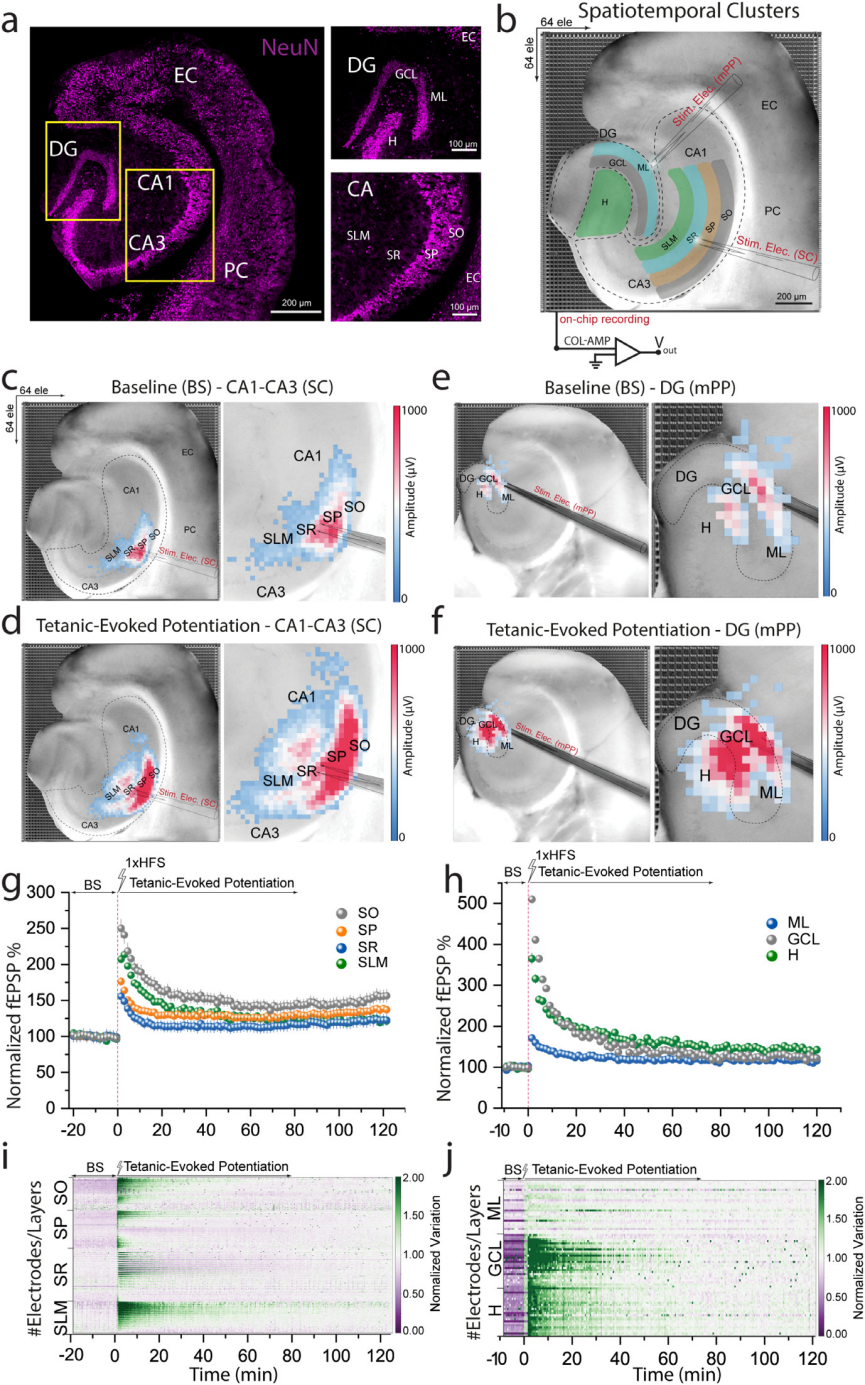
Detailed mapping of network-wide LTP-induced synaptic changes in the hippocampus. (a) Fluorescence images of hippocampal subregions to identify distinct neuronal layers in the network. (b) The platform overlaying functional bioelectrical readouts onto optical images of hippocampal subregions, mapping evoked synaptic activation in various neuronal populations across distinct hippocampal layers (SO, SP, SR, and SLM) in CA1–CA3 network and (ML, GCL, and hilus) in the DG. (c)–(f) Pseudo-color maps showing dynamic representations of baseline and tetanic-evoked potentiation (LTP) for CA1–CA3 and DG networks, highlighting spatial heterogeneity and identifying the exact loci of synaptic strengthening. (g) and (h) Quantification of normalized fEPSPs demonstrating biphasic LTP patterns characterized by immediate and persistent potentiation phases. (i) and (j) Normalized voltage variation color-coded maps for each hippocampal sublayer, revealing layer-specific sensitivity to LTP induction and distinct voltage fluctuations during synaptic potentiation phases.

A key feature of EvoNES is its ability to integrate functional bioelectrical network data with corresponding optical images of the hippocampal subregions. This integration allows EvoNES to function as a dynamic biosensor, precisely mapping layer-specific evoked synaptic activation across diverse neuronal populations, including pyramidal cells and interneurons. By offering bioelectrical feedback in real time, the platform enables high-resolution analysis of cell assemblies and their functional reorganization, which is crucial for understanding the mechanisms of synaptic plasticity in memory processing.^31^ The detailed mapping spans multiple layers, including the stratum oriens (SO), stratum pyramidale (SP), stratum radiatum (SR), and stratum lacunosum-moleculare (SLM) in the CA1–CA3 network, as well as the molecular layer (ML), granule cell layer (GCL), and the Hilus (H) in the DG network^32^ [Figs. 2(a) and 2(b)].

To examine the induction of sequential encoding patterns of network-wide LTP and its effects on synaptic plasticity, we employed a high-frequency tetanic stimulation (HFS) protocol with a sequence of electrical pulses at 100 Hz in 1 s duration. This aims to mimic the natural patterns of neuronal activity that lead to synaptic strengthening, ensuring robust LTP induction. Post-stimulation, we observed enhanced synaptic potentiation across the hippocampal network. The multi-site recordings provided detailed insights into the temporal and spatial dependencies of LTP, revealing complex interactions between the stimulation patterns and LTP induction across different hippocampal regions.

Using pseudo-color maps, part of the advanced biosensing data visualization suite, we dynamically represented baseline (BS) and tetanic-evoked potentiation (LTP) states for both CA1–CA3 and DG networks [Figs. 2(c)–2(f)] and (supplementary material Movies 1 and 2). These maps effectively delineate potentiated states, identifying precise loci of synaptic strengthening within the network and revealing spatial heterogeneity in LTP effects. Notably, previously inactive zones in the baseline state exhibited significant strengthening post-LTP induction [Figs. 2(d) and 2(f)].

We further quantified network-level LTP based on normalized fEPSPs, defining initiation with 40% of baseline synaptic potentiation in the CA1–CA3 [Fig. 2(g)] and DG networks [Fig. 2(h)]. Stimulation of SC and mPP fibers revealed an LTP pattern with two distinct stages: an initial rapid potentiation peaking within the first 5 min, followed by a prolonged potentiation that gradually decayed during the late phase (up to 120 min). This pattern was consistent across all hippocampal subfields, demonstrating the robustness of our stimulation protocol. The quantification mapped spatially evoked responses and provided insights into the temporal dynamics of synaptic strengthening. We also computed the percentage of plasticity post-tetanic stimulation relative to baseline by integrating measurements across immediate, intermediate, and long-term post-tetanic phases to capture sustained synaptic dynamics. This revealed significant network-level plasticity: 82% ± 9.7 in SO, 47% ± 5.3 in SP, 31% ± 5.3 in SR, and 54% ± 3 in SLM within CA1–CA3; and 35% ± 5.6 in ML, 157% ± 8.2 in GCL, and 122% ± 8.4 in H within DG (*p* < 10^−8^ *Kolmogorov–Smirnov test*).

Next, we computed normalized voltage variation color-coded maps for each hippocampal sublayer [Figs. 2(i) and 2(j)] to identify LTP-evoked response dependencies on spatial distribution. These maps revealed subtle voltage fluctuations during synaptic potentiation at the network level, showing distinct variations across different hippocampal layers. Significant voltage changes were observed in SO and SLM of CA1–CA3 regions and GCL in DG throughout the potentiation phases, suggesting a layer-specific sensitivity to LTP induction, consistent with early observations from small-scale electrophysiological recordings.^33^ This analysis underscores the spatiotemporal complexity of synaptic modulation within the hippocampus and its implications for neuronal circuitry, memory formation, consolidation, and retrieval. It reflects the sophisticated mechanisms by which the brain processes and stores information through feature-coding cell assembly repertoire.^15,34^

Our findings support that memory is dynamically shaped by neural activity patterns rather than being a static representation.^35,36^ By combining multiple methodologies^37,38^ into a single, powerful tool, we provide unparalleled resolution for mapping the entire hippocampal network. This opens new avenues for exploring how memories are encoded and manipulated within this crucial brain structure. By characterizing these sequential encoding patterns of network-wide LTP, we gain deeper insights into the neural basis of learning and memory.

Having characterized the sequential encoding patterns across the hippocampal network, we now probe the specific synaptic signatures that emerge from these patterns, utilizing unsupervised classification to unravel the intricate waveform repertoires that define synaptic plasticity.

### C. Unveiling synaptic signatures through unsupervised classification of waveform repertoire

A critical question is how a detailed analysis of layer-based waveform signatures can enhance our understanding of synaptic plasticity and network dynamics in the hippocampus. EPSPs and population spikes (PSs) are hallmark signatures of evoked neural activity and synaptic function within the hippocampus, typically studied through various conventional recording methodologies.^13,39,40^ While these phenomena often serve as proxies for neuronal potentiation and collective neuronal firing, our approach enhanced this understanding by utilizing comprehensive multidimensional data from large-scale recordings. These rich data allowed us to identify complex waveform signatures and their spatiotemporal characteristics, offering a deeper understanding of synaptic interactions in memory processes and neural circuit functionality.^41^ This method facilitated unsupervised analysis of numerous waveform patterns across different layers of the CA1–CA3 and DG networks, both at baseline and following tetanic stimulation.

**FIG. 3.**
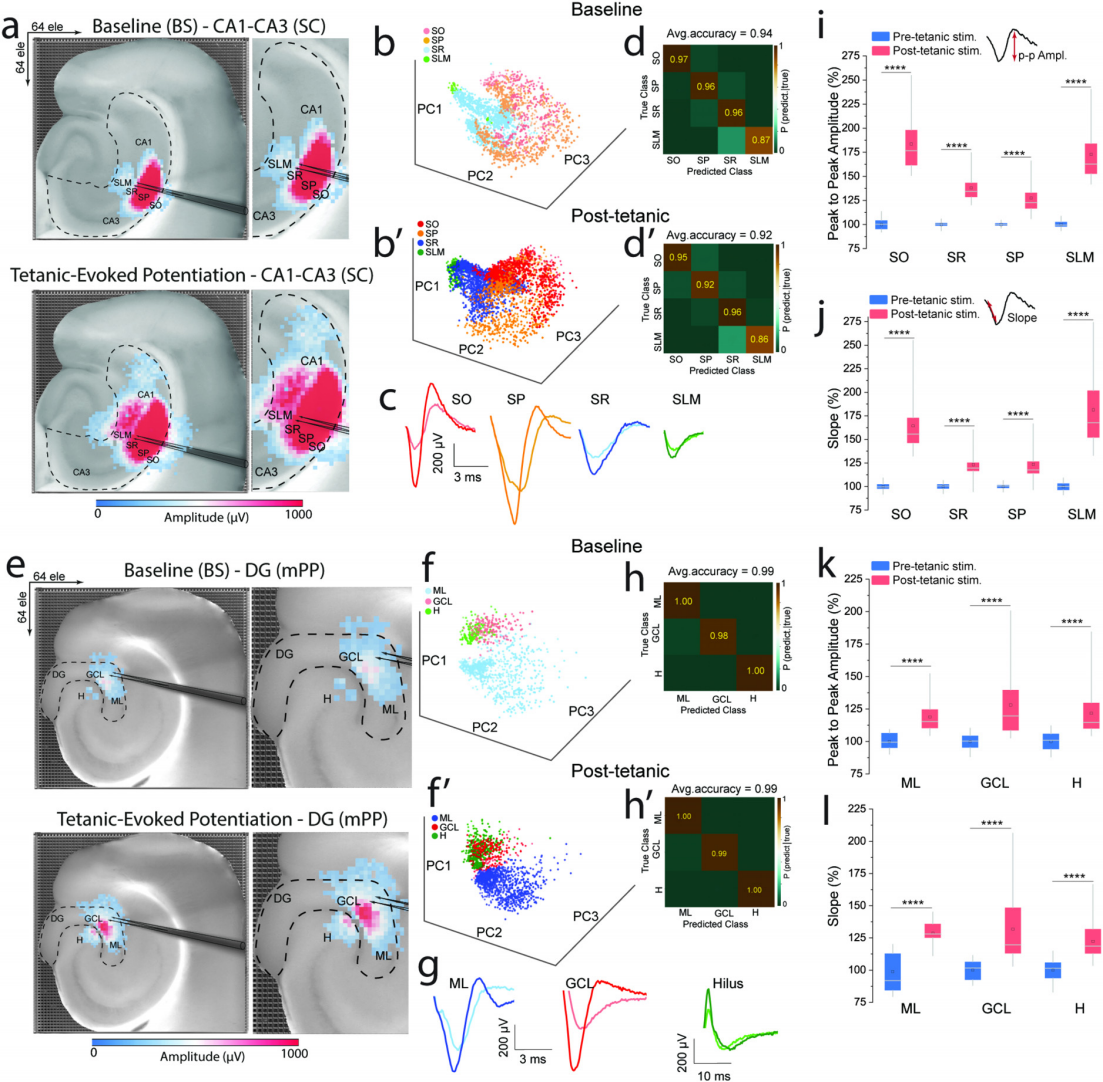
Classification and analysis of network-wide evoked synaptic response patterns. (a)–(c) Principal component analysis (PCA) and k-means clustering identified four distinct EPSP and PS waveform classes in the CA1–CA3 network layers (SO, SP, SR, and SLM) in baseline and tetanic-evoked potentiation phases. (e)–(g) Similar classification in the DG network layers: (ML, GCL, and hilus). (d), (d′), (h), and (h′) Confusion matrices of k-means clustering accuracy scores, showing high precision in classifying and well-defined separation of evoked firing patterns before and after tetanic stimulation in CA1–CA3 and DG networks. (i)–(l) Quantitative analysis of peak-to-peak amplitude and slope features of synaptic responses, demonstrating significant increases across all hippocampal layers post-tetanic stimulation, indicating enhanced neural activation and synaptic plasticity.

Using principal component analysis (PCA) and k-means clustering^24^ (see Sec. IV), we delineated four distinct waveform classes within the CA1–CA3 network, corresponding to SO, SP, SR, and SLM [Figs. 3(a) and 3(c)]. Similarly, we characterized three unique evoked firing patterns in the DG network associated with ML, GCL, and H [Figs. 3(e)–3(g)]. This spatially resolved classification revealed how different hippocampal layers uniquely contribute to synaptic plasticity and functional reorganization.

Temporal analysis of these waveforms showed significant changes post-tetanic stimulation, indicating enhanced neural activation and improved discriminability of neural representations. The increased density and separation of data points in PCA maps post-stimulation [Figs. 3(b), 3(b′), 3(f), and 3(f′)] highlights the selective strengthening of synaptic connections. Classification accuracy was high, with true probability scores of 94% ± 2.3 and 92.2% ± 2.2 for CA1–CA3 networks at baseline and post-tetanic phases, respectively. The DG network showed scores of 99.3% ± 0.6 and 99.6% ± 0.3 for the same phases, confirming the robustness of our classification method [Figs. 3(d), 3(d′), 3(h), and 3(h′)].

We further analyzed the peak-to-peak amplitude and slope features of these waveforms during baseline and post-tetanic phases. This analysis revealed significant increases in both features across all hippocampal layers in response to tetanic stimulation [Figs. 3(i)–3(l)]. These changes underscore the potential of potentiation-based waveform shapes as biomarkers for identifying layer-specific features within the hippocampal circuitry.^42^ Additionally, the observed modifications in neural activity patterns induced by LTP emphasize the dynamic nature of synaptic plasticity and its critical role in information processing and memory formation.

Our findings underscore the importance of integrating spatial and temporal dimensions of firing signatures to fully understand synaptic plasticity and network reorganization associated with LTP,^43^ which underlies activity-dependent modification of neural circuits and engrammed memory formation.^44^ This granular, detailed analysis moves beyond traditional binary indicators, revealing the complex interplay of neuronal activity across various phases and layers of the hippocampus. The identified waveform features, informed by spatiotemporal dependencies, accentuate the capacity to uncover novel biomarkers of synaptic performance and neural circuit integrity.

With a clearer understanding of the potentiation-based waveform signatures within hippocampal layers, we next focus on mapping the spatiotemporal dynamics of evoked synaptic transmission, providing insight into how these dynamics facilitate complex learning and memory processes across the network.

### D. Mapping spatiotemporal dynamics of network-wide evoked transmission

Understanding the spatiotemporal dynamics of synaptic activation within the hippocampus is crucial for elucidating the mechanisms underlying learning and memory. Despite the established roles of neuronal firing patterns and synaptic plasticity in these processes, comprehensive mapping of these dynamics remains challenging.^45,46^ Spike-timing-dependent plasticity (STDP) highlights the importance of temporal precision in learning mechanisms.^47^ Additionally, the spatial organization of synaptic activation is vital for efficient information processing in the hippocampus, supporting spatial navigation and memory integration.^48,49^ Despite advances, a complete understanding of these spatiotemporal dynamics poses significant challenges, representing a critical frontier in learning and memory research.

**FIG. 4.**
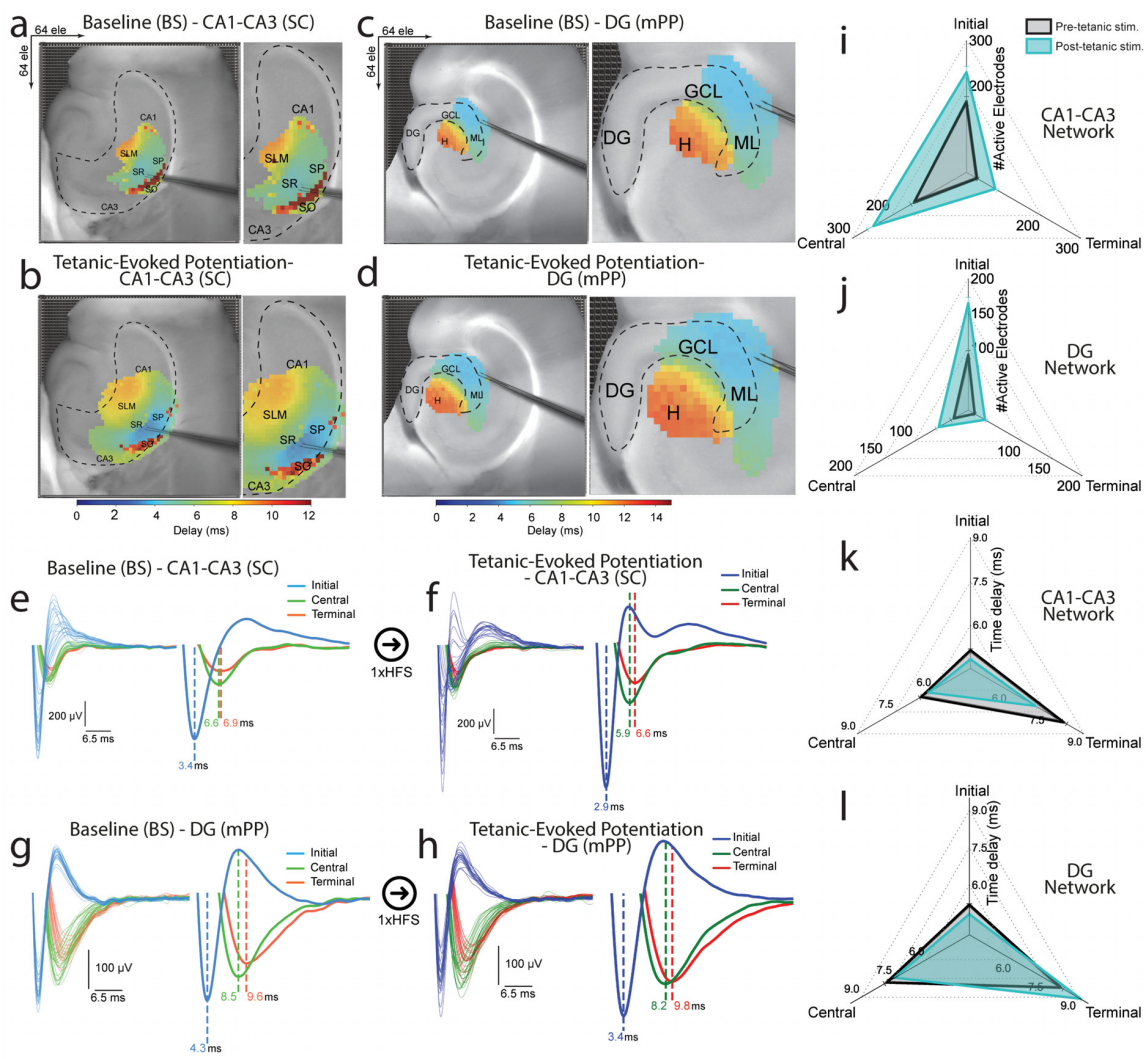
High-order temporal and spatial dynamics of network synaptic activation. (a)–(d) Pseudo-color latency maps showing the spatial distribution of synaptic responses in the CA1–CA3 and DG networks at baseline and post-tetanic phases. These maps categorize synaptic events into initial, central, and terminal clusters based on their timing relative to the stimulating electrode. (e)–(h) Temporal clustering of synaptic responses illustrates the precise activation timing within hippocampal layers, highlighting significant changes in response patterns post-tetanic stimulation. (i)–(l) Quantitative analysis reveals a substantial reduction in response latency and increased active electrodes after tetanic stimulation, indicating enhanced synaptic efficiency and network restructuring. Notably, new firing electrodes emerge in previously silent regions, underscoring the dynamic reorganization of synaptic networks.

To investigate these dynamics, we characterized extracellular synaptic responses by their post-stimulation latencies. We clustered peak time indices from processed evoked waveforms into initial, central, and terminal groups. We established correlations between the spatial distribution of synaptic events relative to the stimulating electrode and their temporal characteristics across CA1–CA3 and DG networks in both baseline and post-tetanic phases, as illustrated in the pseudo-color latency maps [Figs. 4(a)–4(d)]. The time-based waveform clusters showed distinct timing of synaptic responses relative to the stimulation point [Figs. 4(e)–4(h)]. We computed time delay dynamics and tracked the distribution of activated electrodes in initial, central, and terminal clusters from baseline to post-tetanic phases, leveraging the advanced biosensing capabilities of the EvoNES platform. Notably, in the post-tetanic phase, we observed the emergence of new firing electrodes, indicating the activation of previously silent or less active regions within the network. This demonstrates the platform's unique ability to uncover latent neuronal functionality, which could align with the principles of associative LTP.^50^ This activation reflects a mesoscale reorganization consistent with the functional restructuring of synaptic networks following LTP induction, including the recruitment and strengthening of circuit-level responses across hippocampal layers. Additionally, this phase was marked by a substantial reduction in synaptic response latency, suggesting increased synaptic efficacy and more efficient signal propagation across the network. While these dynamics are indicative of plasticity-induced changes, they are inferred from extracellular population recordings and do not imply direct observation of synaptic ultrastructure or molecular remodeling.

A quantitative analysis of the time delay and the number of active electrodes from multiple recordings confirmed a notable decrease in time delay (i.e., faster induction) and an expansion of the activated zone post-tetanically, indicating enhanced spatiotemporal efficacy of synaptic activity [Figs. 4(i)–4(l)]. The activation of new firing electrodes, particularly in regions previously classified as silent or less active, marked a significant restructuring of synaptic networks. This was evident across CA1–CA3 (SO, SR, SP, and SLM) networks, with 1.5, 1.8, and 2.25-fold increases in initial, central, and terminal layers, respectively. Similarly, the DG (ML, GCL, and hilus) showed 1.8-, 1.7-, and 1.9-fold increases in initial, central, and terminal clusters, respectively, highlighting the potentiation of synaptic activity [Figs. 4(i) and 4(j)]. The normalized percentage change in temporal delay from baseline to post-tetanic phases [Figs. 4(k) and 4(l)] was reduced by 6% ± 0.3, 4.8% ± 0.4, and 13.8% ± 0.25 in the initial, central, and terminal clusters of the CA1–CA3 network, respectively; and by 7.3% ± 0.45 and 4.8% ± 0.3 in the initial and central clusters of the DG network, while the terminal cluster of the DG exhibited an increased delay of 10.8% ± 0.42.

Our temporal mapping in the CA1–CA3 hippocampal network revealed the sequence of responses to tetanic stimulation, beginning with the SR, where direct excitatory inputs from the CA3 area are first integrated. This initial response is followed by activity in the SP, where these inputs are processed into neuronal outputs. Subsequently, the SO and SLM respond, reflecting their roles in modulating and integrating the network's output through their complex inputs and indirect pathways. This unique orderly progression from initial integration (SR) through neuronal processing (SP) to modulation (SO and SLM) emphasizes the importance of understanding the sequence of synaptic activation and plasticity mechanisms in the hippocampus. Similarly, we mapped the temporal dynamics before and after tetanic stimulation in the DG layers, showing the sequence of synaptic activation and plasticity induction from the input layer (ML) through the processing layer (GCL) to the modulatory circuit (hilus). This progression underlines the complexity of information processing and synaptic modification in response to high-frequency stimulation.

Our findings underscore the intricate interplay between the spatial positioning of synaptic activation and their temporal responsiveness to neural stimulation. This spatiotemporal framework enhances our understanding of synaptic organization in hippocampal layers and the dynamic processes of LTP and network adaptation. These insights highlight the hippocampus's role in memory and learning by showing how changes in synaptic efficiency and new cell assembly activations contribute to cognitive functions. Our results may offer empirical evidence of how the hippocampus processes latent information, particularly in the DG–CA3 circuitry,^51^ and how it dynamically reorganizes and encodes new information in response to external stimuli and experiences.

Building upon the detailed understanding of the spatiotemporal dynamics of synaptic transmission, Sec. II E will explore the contributions of mesoscale transmembrane generators to these dynamics, thereby advancing our comprehension of the complex synaptic interactions within the hippocampal network.

### E. Identifying mesoscale transmembrane generators of sequential network-level LTP patterns

The relationship between electrical potential distributions and their corresponding sink and source currents in extracellular synaptic activation reveals essential insights into the principles of transmembrane generators and functional cell assemblies in the hippocampal circuit.^52^ Variations in the magnitude and duration of these currents during synaptic events modulated by LTP provide a deeper understanding of synaptic transmission efficacy and the activation of complex neuronal assemblies with complex patterns coordinated across space and time. Recording methodologies with high spatiotemporal resolution are essential for clarifying these mesoscale synaptic dynamics and advancing our understanding of learning and memory processes.

**FIG. 5.**
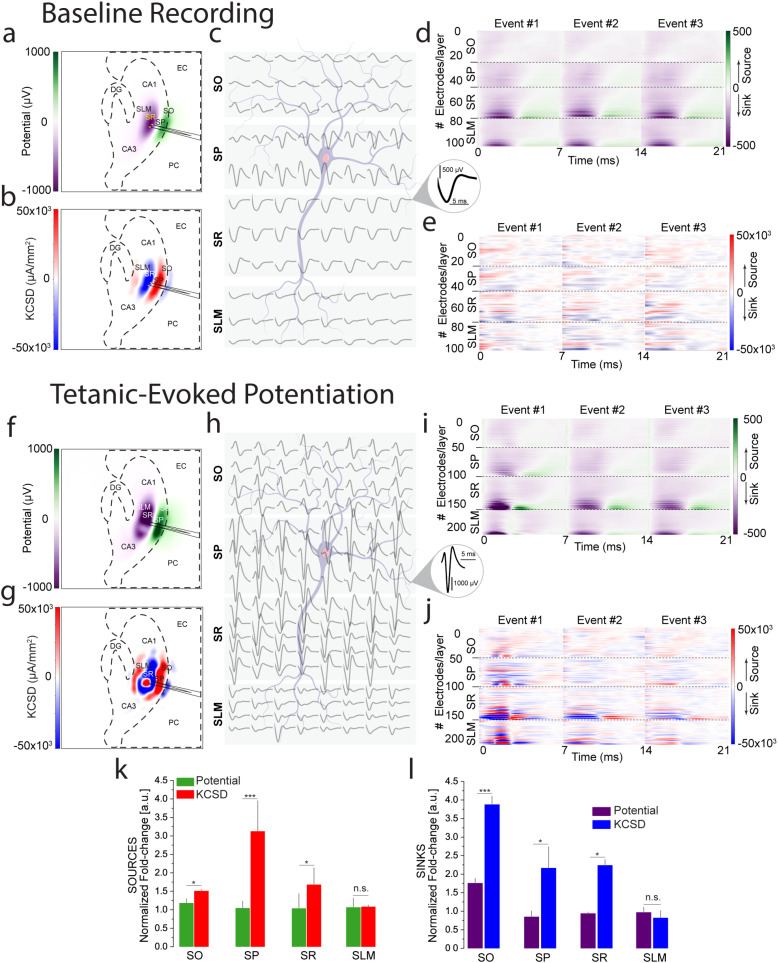
Mesoscale mapping of network synaptic activity and connectivity dynamics. (a), (b), (f), and (g) Bidimensional maps showing potential-based and kernel current source density (kCSD) representations of synaptic activity in the CA1–CA3 network under baseline and post-tetanic stimulation conditions. (c) and (h) Extracellular potential signatures across different hippocampal layers, illustrating network-level synaptic dynamics and connectivity changes from baseline to tetanic-evoked potentiation. (d), (e), (i), and (j**)** Time-resolved synaptic activity maps, with the x-axis representing the timing of evoked events and the y-axis corresponding to firing electrodes within each layer. These maps provide a detailed view of sinks (associated with incoming EPSPs) and sources (related to synchronous neuronal firing). (k) and (l) Comparison of quantified potential sources and sinks against those identified in the kCSD maps, highlighting the kCSD analysis's superior precision in localizing synaptic events. This approach enhances our understanding of synaptic transmission and network dynamics in response to LTP, revealing intricate differences in sink and source values both at baseline and post-tetanic conditions.

Here, we constructed two distinct spatiotemporal bidimensional maps—potential-based and kernel current source density (kCSD)^53^—across a 64 × 64 grid that mirrors the HD-CMOS-MEA's geometry. This approach enabled the quantification of dynamic shifts in transmembrane current flow across hippocampal sublayers (SO, SP, SR, SLM) in the CA1–CA3 network, during baseline and post-tetanic conditions [Figs. 5(a)–5(h)]. The potential-based maps provided high-resolution visualization of extracellular field potentials, while the kCSD maps enabled precise identification of layer-specific differences in current sink–source distributions and polarity. Following post-tetanic stimulation, we observed a prominent amplification of inward sinks in SR—consistent with enhanced excitatory input onto apical dendrites—alongside more sharply localized and spatially redistributed sources in SP, indicative of strengthened somatic integration. In SLM, potentiation led to sharpened current polarity and localized source–sink coupling, potentially reflecting changes in return currents or attenuation of distal inputs. In SO, we detected a broad amplification and slight spatial expansion of sink activity, suggesting enhanced recruitment of perisomatic and basal dendritic compartments. These layer-specific synaptic signatures reflect subfield-dependent mechanisms of plasticity reorganization, capturing how network-level LTP is integrated across hippocampal laminae. The high spatial resolution of our CMOS-MEA platform enables direct, large-scale current mapping—without relying on invasive or impractical dense electrode configurations—offering a precise electrophysiological readout of laminar-specific plasticity. These findings define how distinct hippocampal layers reorganize in response to potentiation, establishing a scalable systems-level approach to track spatially resolved synaptic remodeling.

Moreover, we organized the evoked synaptic data pre- and post-tetanic stimulation, aligning the x-axis with the timing of each evoked event and the y-axis with specific firing electrodes. This arrangement, coupled with computed potential-based and kCSD-derived source/sink generator data, provided a dynamic, spatial, and temporal view of layer-specific synaptic activity patterns [Figs. 5(d), 5(e), 5(i), and 5(j)]. While potential maps showed only a binary change from sink to source, the kCSD approach demonstrated more subtle and complex dynamic changes during LTP induction. When comparing the three selected events along the potentiation timeline—from Event #1 to Event #3 at the final stage when potentiation returned to baseline—the kCSD illustrated a far more intricate behavior during potentiation, which became increasingly complex and enhanced after tetanic stimulation. This dual-mapping enabled a layered, time-resolved representation of synaptic patterns, highlighting sinks (magenta/blue) associated with incoming EPSPs and sources (green/red) related to population spikes of synchronous neuronal firing. These findings capture nuanced synaptic changes, providing deeper insights into the mechanisms underlying synaptic plasticity and memory encoding at the network level.

Comparative analysis of quantified potential sources and sinks against those identified in the kCSD maps underscored these pronounced differences [Figs. 5(k) and 5(l)]. The potential-based analysis provided a general view of synaptic potentiation yet struggled to accurately resolve the spatial origin of synaptic events, which made it challenging to differentiate between EPSP and PS responses associated with precise sink–source generators. In contrast, the kCSD analysis offered a more precise localization of synaptic events, providing clearer insights into the underlying synaptic dynamics. Despite challenges in comparing these analyses due to their fundamentally different informational nature, their combined use is crucial for a comprehensive understanding of synaptic activity and connectivity. This integrative quantification of extracellular potentials and reconstructed transmembrane current flow refines our capacity to resolve layer-specific synaptic integration, offering a mechanistic dissection of how network-wide potentiation is orchestrated across distinct hippocampal sublayers. These findings consolidate the role of mesoscale current mapping in revealing the principles of synaptic coordination underlying memory encoding. Building on these advanced insights, Sec. II F will examine how aging modifies these synaptic processes, comprehensively analyzing the variable impacts across different hippocampal layers.

### F. Network-resolved LTP and synaptic transmission in aging

Studying synaptic dynamics at the network level in aging circuits is essential for understanding how aging affects learning and memory. Fully grasping the influence of aging on hippocampal networks demands comprehensive, simultaneous monitoring of synaptic activity throughout the hippocampus, manifested through LTP induction. Methodological limitations have restricted a deeper insight into these age-related synaptic modifications.

Here, we utilized EvoNES to explore how aging alters synaptic function by examining layer-specific responses to LTP induction within the CA1–CA3 network. This approach enabled a detailed study of synaptic efficacy and neuronal deactivation sequences, providing insights into how intrinsic aging affects the circuitry.

**FIG. 6.**
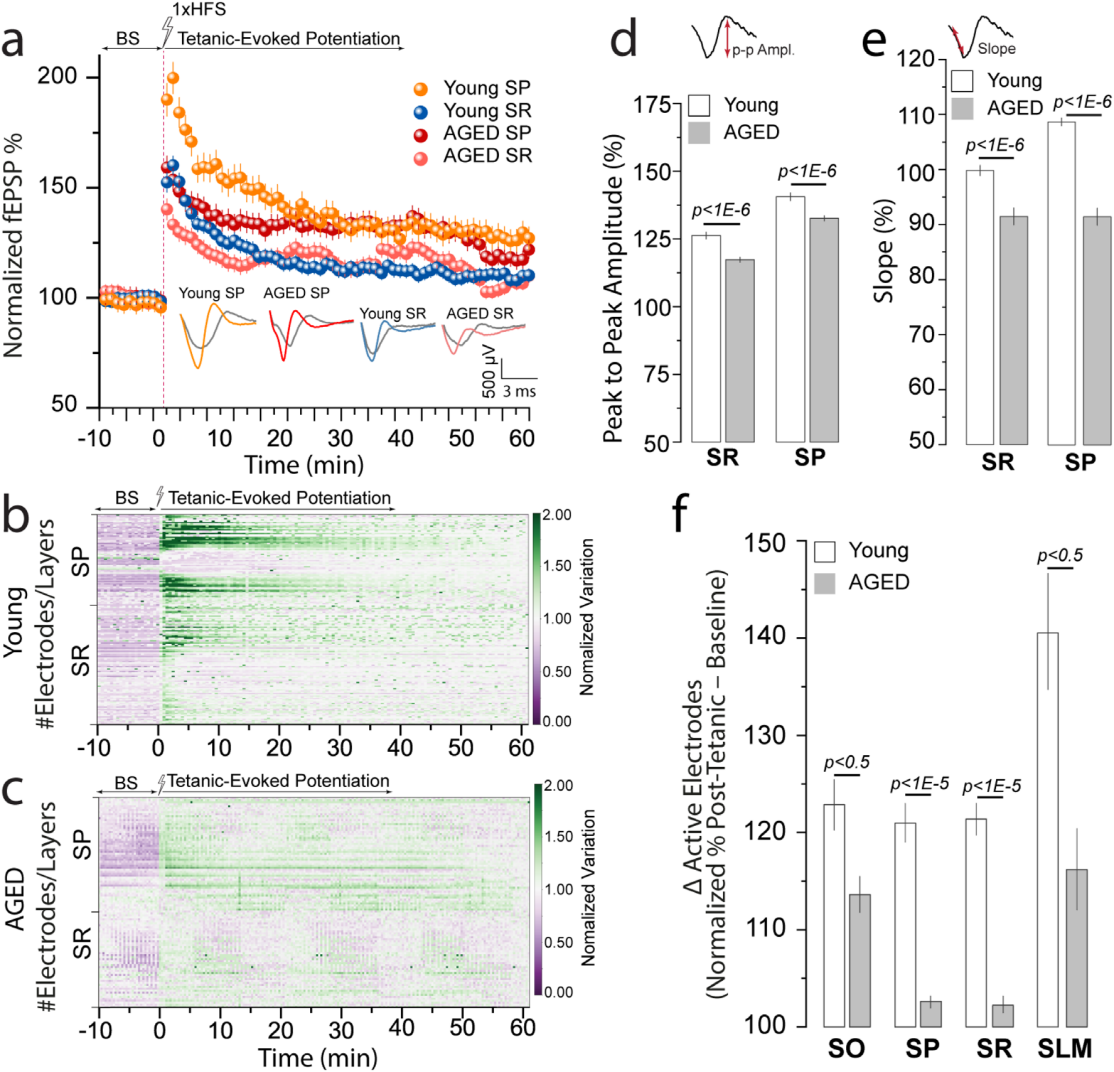
Impact of aging on network-level synaptic plasticity and LTP in the hippocampus. (a) Comparison of normalized network-based fEPSPs in CA1–CA3 layers (SP and SR) between aged and young standard mice, showing a significant decline in synaptic plasticity in the aged group. Also, this decline is identified in the layer-specific EPSP and PS patterns under the potentiation curve. (b) and (c) Normalized voltage variation color-coded maps illustrating spatial distribution of LTP-evoked responses across hippocampal sublayers in aged vs control young groups, highlighting intricate layer-specific quiescence and potentiation dependencies. (d) and (e) Quantitative waveform analysis of synaptically evoked activity in SP and SR layers, revealing aging-related changes in amplitude and slope features during tetanic-evoked potentiation phases. (f) Normalized difference in active electrode counts (post-tetanic %–baseline %) across CA1–CA3 layers, showing significantly smaller increases in firing activity in aged mice compared to young controls. These results reflect aging-related deficits in hippocampal network responsiveness and plasticity.

Upon establishing a stable baseline, a single HFS session triggered LTP across the entire network in aged mice. The fEPSP was quantified within the SP and SR layers of the CA1–CA3 network compared to the control group of young adult mice [Fig. 6(a)]. Notably, we observed a marked decline in synaptic plasticity in aged circuits compared to younger counterparts, with significant reductions in the fEPSP measurements: 38% ± 4.7 in SP and 21% ± 3.9 in SR, contrasted with 49.9% ± 5.9 and 24.8% ± 3.5, respectively, in young controls (*p* < 0.01, *Kolmogorov–Smirnov test*).

Further, normalized voltage variation color-coded maps revealed how LTP-evoked responses depend on the spatial distribution across hippocampal sublayers SP and SR in the aged vs young adult groups [Figs. 6(b) and 6(c)]. Moreover, a quantitative waveform analysis of synaptically evoked activity in layers SP and SR revealed critical insights into the amplitude and slope features of spatially identified responses in the tetanic-evoked potentiation phases [Figs. 6(d) and 6(e)], highlighting the impact of aging on network synaptic functionality. These results support the presence of a layer-specific potentiation profile across hippocampal circuits, with aging modulating the magnitude and spatial distribution of this response. EvoNES enables reliable detection of such laminar-specific alterations, providing a framework for the prospective identification of electrophysiological biomarkers—such as layer-dependent waveform shifts and electrode activation patterns—that may serve as early indicators of network vulnerability in aging and neurodegenerative conditions.

Our comprehensive examination also encompassed other layers in the CA1–CA3 network (i.e., SO and SLM), where we identified changes in neuronal firing associations evidenced by a reduced count of active electrodes from baseline to post-tetanic phases [Fig. 6(f)]. This decline of spatially distributed firing zones likely reflects diminished recruitment of functional cell assemblies and reduced temporal precision in their evoked responses within aged hippocampal circuits, indicative of compromised synaptic coordination. Although our approach does not resolve single-cell assemblies, the mesoscale resolution of our recordings captures collective neuronal dynamics, enabling inference of ensemble-level activity patterns. These findings provide a robust, data-driven readout of aging-related disruptions in the spatial and temporal structure of synaptic responses, consistent with impairments in Hebbian-like plasticity mechanisms.^54^

In summary, our results delineate an altered landscape of synaptic plasticity across aging hippocampal networks, supported by reductions in potentiation amplitude, shifts in waveform features, and deactivation of functional firing zones. Together, these changes capture circuit-level reorganization associated with aging and establish a framework for future studies on hippocampal vulnerability and plasticity resilience in age-related cognitive decline.

## III. DISCUSSION

Our study marks a significant advancement in leveraging biosensing technologies to unravel the spatiotemporal dynamics of synaptic plasticity and LTP within the hippocampal circuit at the network level. By integrating high-resolution CMOS-based biosensors with advanced computational tools and bidirectional stimulation and recording capabilities, EvoNES provides unparalleled insights into the dynamic interplay of hippocampal subfields during synaptic potentiation and LTP dynamics. With the transformative role of EvoNES as a next-generation biosensing platform, we address critical limitations of traditional methods, including sharp electrode recordings, patch-clamp techniques, extracellular field measurement, conventional MEAs, and optical imaging methodologies. This biosensing-enabled advancement facilitates precise, scalable, label-free, comprehensive mapping of synaptic dynamics at the network level—essential for decoding the complex cellular interactions that underpin learning and memory processes. A fundamental strength of this study is its capacity to mimic natural neuronal activities at the network level through direct electrical tetanic stimulation, providing a controlled *ex vivo* environment that closely mirrors *in vivo* conditions. The mesoscale modeling of real-time synaptic dynamics across hippocampal layers offers a novel systems-level view of how coordinated synaptic activity may support memory-relevant processing, aligning with established frameworks of engram dynamics and memory trace formation in distributed networks.^36,55^

The waveform repertoire extracted across hippocampal lamina using EvoNES further substantiates this systems-level view by linking specific signal classes to anatomically and functionally distinct sublayers. In CA1–CA3, signals in SO reflect multiphasic responses driven by local interneurons and recurrent collaterals; SP captures population spikes from synchronized pyramidal cell firing; SR signals denote dendritic EPSPs from Schaffer collateral inputs; and SLM reflects distal EPSPs from entorhinal projections. Similarly, in the DG, ML waveforms indicate perforant path activation, GCL reflects synchronized somatic granule cell activity, and hilus signals integrate broader excitatory and inhibitory inputs. While these waveform classes do not resolve single-cell firing, they serve as reproducible mesoscale patterns of synaptic dynamics allowing a high-resolution functional map of input-specific recruitment during LTP.

Our findings highlight how spatial and temporal dependencies critically influence synaptic efficacy, revealing intricate modulatory mechanisms that shape learning and memory. These insights affirm the significance of large-scale neural dynamics in orchestrating memory formation and retrieval processes, showcasing the dynamic nature of neural networks.^56,57^

We have elucidated the functional dynamics of the hippocampus, emphasizing the crucial role of sequential activation from the mPP to DG and from the SC to the CA regions. This supports the concept of the hippocampus as a dynamic indexer, intricately linking memories across the cortex through well-coordinated cell assemblies.^58^ Our findings on the critical role of network-level LTP in memory encoding within specific layers significantly advance our understanding of complex neural mechanisms. These observations underscore the importance of studying network-level synaptic processes to unravel the intricacies of memory formation. The use of kCSD analysis in this study added a powerful spatial dimension to our interpretation of hippocampal network responses. By reconstructing source–sink distributions from high-density extracellular signals, kCSD enabled a fine-grained visualization of layer-specific synaptic activation following LTP induction. Unlike raw voltage traces, these spatially resolved maps captured shifts in transmembrane current flow, revealing dynamic reorganization of active pathways across hippocampal subregions. This analytical approach complements our waveform and latency analyses by confirming not only the recruitment of additional network elements but also their spatial localization, thus reinforcing our interpretation of plasticity-induced reweighting at the circuit level.

Leveraging the bioelectrical sensing precision of EvoNES, the study provides empirical evidence for representational drift, a mechanism suggesting the adaptability of memory systems essential for the ongoing updating and retrieval of memories.^59^ This phenomenon could underlie the evolution of firing patterns of neuronal populations over time as a transition from baseline activity patterns to those induced post-tetanically. This adaptability is crucial for incorporating newly acquired information into long-term memory, enhancing the flexibility and resilience of memory networks.^60^ Our findings may also support the memory allocation hypothesis by demonstrating that excitability-driven recruitment of neuronal populations occurs in a layer-specific manner during LTP induction.^61^ While we did not analyze individual cell assemblies, the network-wide signatures of LTP captured through our mesoscale approach reveal selective patterns of potentiation and suppression (e.g., in young vs aged groups), reflecting dynamic shifts in excitability across hippocampal layers. Notably, observed deactivations in certain conditions may reflect differential thresholds for recruitment, further emphasizing the non-uniform, activity-dependent mechanisms by which specific neuronal populations contribute to plasticity and memory encoding.

While this study focused on network-wide LTP, LTD also plays a vital role in memory processes by fine-tuning synaptic connections and preventing memory generalization, thus contributing to spatial learning and information encoding.^62^ Although our findings emphasize network-level LTP, the platform is equally equipped to investigate LTD dynamics. Its bidirectional stimulation and high-resolution sensing capabilities make it a comprehensive tool for examining the complementary roles of synaptic potentiation and depression in memory encoding and storage.

In the context of aging, our data reveal how synaptic plasticity is affected, with certain hippocampal layers showing decreased synaptic transmission and LTP induction in the aged compared to younger counterparts. These age-related changes offer potential biomarkers for cognitive decline, providing insights into the synaptic foundations of aging within the hippocampus and implications for early detection of neurodegenerative conditions such as Alzheimer's disease.^31,63^

The foundational research conducted here has successfully highlighted key aspects of synaptic dynamics and plasticity, setting the groundwork for a range of exciting future investigations that extend beyond the initial scope of our study. These include exploring metaplasticity,^64^ the interconnectedness of cell assemblies,^65^ and the broader cellular mechanisms that underpin synaptic plasticity, such as NMDA receptor dynamics and the role of neuromodulators.^13^ Future studies could bridge the gap between neuroscience and practical brain-inspired technological applications, such as machine learning and neuromorphic computing, leveraging our findings to enhance the learning capabilities of artificial systems.^66^ This comprehensive approach not only extends our initial findings but also paves the way for groundbreaking interdisciplinary research that merges neuroscience with technological innovations, setting a new course for future explorations that harness a deep understanding of neural dynamics, promising to revolutionize both theoretical and practical aspects of neuroscience.

## IV. METHODS

### A. Animals and housing conditions

To investigate network synaptic transmission and LTP, experiments were conducted on C57BL/6J female mice aged 12 weeks (young adult group) and 56 weeks (aged group), obtained from Charles River Laboratories, Germany. All procedures complied with relevant European and national regulations (Tierschutzgesetz) and received approval from the local authority, Landesdirektion Sachsen (Approval No. 25-5131/476/14).

### B. Acute hippocampal brain slices preparation

Mice were anesthetized with isoflurane before decapitation, and slices were prepared according to our previous studies.^24–26^ The brain was carefully removed from the skull and placed in an ice-chilled high-sucrose cutting solution before slicing. The brain was securely placed in a custom-made agarose-based container and then affixed to the cutting plate. Horizontal slices of 300 *μ*m thickness were prepared using Leica Vibratome VT1200S (Leica Microsystems, Germany). Slices were cut at 0–2 °C in a high-sucrose artificial cerebro-spinal fluid (aCSF) solution saturated with 95% O_2_ and 5% CO_2_ (pH = 7.2–7.4) containing in mM 250 sucrose, 10 glucose, 1.25 NaH_2_PO_4_, 24 NaHCO_3_, 2.5 KCl, 0.5 ascorbic acid, 4 MgCl_2_, 1.2 MgSO_4_, 0.5 CaCl_2_. Next, hippocampal-cortical slices were incubated for 45 min at 32 °C and then allowed to recover for at least 30 min at room temperature. The recording aCSF solution used for electrically evoked synaptic response recordings contained in (mM): 127 NaCl, 3.5 KCl, 1.25 NaH_2_PO_4_, 26 NaHCO_3_, 10 glucose, 1 MgSO_4_, 2.5 CaCl_2_, and was saturated with 95% O_2_ and 5% CO_2_.

### C. Extracellular evoked synaptic responses and network-level LTP in the EvoNES platform

The EvoNES platform, a next-generation biosensing system, facilitates stimulus-responsive biosensing functionality through its customized HD-CMOS-MEA biosensors (3Brain AG, Switzerland). With 4096 electrodes, each separated by a 42 *μ*m pitch covering an active sensing area of approximately 7 mm^2^, this biosensor system is uniquely engineered for high-resolution, large-scale neural activity mapping. To interface brain tissue slices with the electrodes, we employed a custom-designed platinum harp positioned directly above the tissue to ensure optimal contact. For sustained tissue viability and consistent experimental conditions, we implemented a temperature-regulated perfusion system. This system continuously supplied aCSF to the tissue–electrode interface at a flow rate of 4.5 ml/min, maintaining a constant temperature of 37 °C throughout the experiments. All extracellular synaptic recordings were performed at 14 kHz/electrode sampling frequency. To facilitate detailed studies on large-scale evoked synaptic responses and LTP, we enhanced the EvoNES platform with a precision zero-drift triple-axis micromanipulator system (SENSAPEX, Finland). Additionally, a bipolar electrode (70% platinum, 30% iridium) was utilized (World Precision Instruments, Germany). The electrode featured a tip diameter of 3 *μ*m, an outside diameter of 0.356 mm, and a length of 51 mm, with a tip separation of 125 *μ*m at a nominal impedance of 2 MΩ, enabling focal or broader pathway activation depending on stimulation parameters and positioning.

### D. EvoNES stimulation-recording protocol

To generate sequential fEPSPs, the bipolar electrode, functioning as a core component of the biosensing workflow, was strategically positioned either in the mPP of the DG or in the SR of the CA region to stimulate the SC pathway. The precision of electrode placement, ensured by the platform's zero-drift manipulator, underscores the advanced biosensing capabilities for targeted neural stimulation and high-fidelity response acquisition. A monophasic constant voltage pulse was applied with a pulse half-width ranging from 70 to 140 *μ*s, where evoked responses were simultaneously monitored. To calibrate the optimal stimulation intensity, we established an input/output curve by incrementally increasing the stimulation in steps of 10 *μ*A from 20 to 130 *μ*A at 30 s intervals, maintaining the same pulse half-width. The intensity that evoked 60% of the maximum fEPSP slope was identified and used for both baseline and tetanic-evoked potentiation phases. This selected intensity was automatically applied every 30 s using test pulses at 0.033 Hz until a stable baseline recording was achieved for 10–20 min. LTP was induced via a single high-frequency stimulation (HFS) train at 100 Hz, with a 10 ms interval and the same pulse half-width. We recorded evoked responses in the hippocampal regions up to 2 h. Significant LTP was documented if the responses post-HFS during the tetanic-evoked potentiation phase were at least 40% greater than those during the BS phase.^16^ After recording the evoked synaptic responses, images of brain slices coupled to the HD-CMOS-MEA during network stimulation were captured by an optical modular stereomicroscope (Leica Microsystems, Germany). This also facilitated the analysis of the spatial organization and clusters of the tissue relative to the electrode layout.

### E. Data analysis

The data analysis pipeline, integral to the EvoNES biosensing platform, developed custom-designed Python scripts to process and extract spatiotemporal patterns of neural activity. This computational framework complements the biosensor's high-resolution data acquisition, enabling the identification and quantification of subtle electrophysiological changes across the hippocampus. Any package add-ons are cited accordingly.

#### 1. Functional-structural clustering for spatiotemporal analysis

To analyze evoked synaptic responses and LTP across specific hippocampal layers, we assigned firing electrodes to designated regions of the hippocampus. We generated topographical pseudo-color maps of large-scale evoked firing patterns from our recordings, which were superimposed on structural images of the hippocampus. Additionally, images captured with a light microscope were overlaid on the layout of the HD-CMOS-MEA to facilitate precise localization using Brainwave software (3Brain AG, Switzerland). Electrodes were systematically categorized into clusters corresponding to distinct structural landmarks within the hippocampal slice. These clusters encompassed six critical regions involved in hippocampal circuitry: the molecular layer (ML), granule cell layer (GCL), and hilus (H) within the DG, as well as the stratum oriens (SO), stratum pyramidale (SP), stratum radiatum (SR), and stratum lacunosum-moleculare (SLM) in the CA regions.

#### 2. Automated classification of waveform signatures

To systematically analyze the waveform signatures of evoked synaptic responses across distinct hippocampal layers, we employed an automated classification system integrating Principal Component Analysis (PCA) and means clustering.^24,67^ The methodology was applied to elucidate the complex waveform shapes recorded from hippocampal circuits, specifically targeting responses from DG and CA layers following stimulation. Initially, PCA was employed to reduce the dimensionality of the data derived from sequential distinct stimulation events, capturing the primary features of the waveform shapes that signify different types of neural activity and focusing on the most informative aspects of the waveforms and their variance across samples. Subsequently, the means clustering algorithm was applied to these PCA-reduced data to categorize the waveforms into specific clusters.^24^ Each cluster was associated with evoked responses from particular layers within the hippocampus: ML, GCL, and H in the DG and SO, SP, SR, and SLM in the CA regions. This classification facilitated the identification of distinct waveform patterns corresponding to different types of synaptic activity (i.e., EPSPs and PS) influenced by either mPP or SC pathway stimulations. To quantify the effectiveness of our classification, we computed the accuracy through the analysis of a confusion matrix. The average true positive rate was assessed by calculating the mean diagonal probability of the matrix, which compares the predicted class labels to the actual class labels. This probability ranges from 0 to 1, where a higher value indicates better accuracy and class specificity in the classification. The results from the confusion matrix were compelling, demonstrating well-defined separation and high accuracy in distinguishing between waveform classes associated with specific hippocampal layers. This procedure was partly implemented using the Scikit-learn 1.0.2: Machine Learning in Python and is available on GitHub (https://github.com/scikit-learn/scikit-learn/blob/main/sklearn/decomposition/_pca.py).

#### 3. Framework for temporal clustering of network-based evoked synaptic responses

To analyze the temporal dynamics of evoked synaptic responses within the hippocampal layers, we employed a refined spatiotemporal clustering algorithm focusing on peak time delays of electrically evoked synaptic responses. This approach enabled us to discern distinct clusters that reflect coordinated changes in synaptic timing, indicative of underlying synaptic plasticity and functional connectivity. It also allowed us to analyze evoked events from different experiments and animals and compare several slices' responses regardless of the minor differences in the position of the external bipolar electrode to the mPP or SC region of stimulation interest. This involved applying a fourth-order Butterworth bandpass filter (1–500 Hz) to eliminate noise and artifact interference, ensuring the preservation of waveform integrity. Following this, peak detection algorithms were employed to identify the exact timing of each peak within the waveform, which was crucial for the subsequent temporal clustering. We then categorized these identified peaks into three temporal clusters—initial, central, and terminal—based on their peak time delays from the stimulus onset. This categorization was achieved through hierarchical clustering, which organized the peaks according to their temporal proximity to the stimulation. This allowed us to discern patterns in the timing of synaptic activations across different hippocampal layers. For validation, the robustness of the clustering process was assessed by analyzing the consistency of temporal groupings across multiple experimental sessions. Additionally, we examined the intra-cluster and inter-cluster variability to ensure the reliability of our temporal categorization. The final step involved visualizing and quantitatively analyzing these temporal clusters. We created pseudo-color latency maps to depict the distribution of synaptic events spatially and temporally, highlighting how different hippocampus regions responded over time to the stimulus. This visual representation and statistical analysis of changes in firing timings and electrode activations from baseline to post-tetanic phases provided deep insights into synaptic activation dynamics and synaptic plasticity's underlying mechanisms. Through this methodology, we captured and elucidated the intricate temporal patterns of synaptic responses fundamental to understanding hippocampal function in learning and memory.

#### 4. Kernel current source density (kCSD) analysis

To elucidate the sources and sinks of synaptic activity within the hippocampal layers using EvoNES, we employed the kCSD analysis,^27^ facilitated by the open-source kCSD-python package (https://github.com/Neuroinflab/kCSD-python/blob/master/kcsd/KCSD.py).^68^ This method is particularly well-suited for HD-CMOS-MEA because it can handle arbitrary electrode distributions, so it remains stable in case of contact malfunction. It uses regularization to reduce noise effects on analysis and accounts for differences in conductivity between tissue and the saline covering the brain slice.^69^ The kCSD method employs a smoothing kernel to estimate the potential everywhere on the slice and a corresponding cross-kernel to move from the potential to the current source density.^27^ These kernels encapsulate the conductive properties of the medium and geometry of the system.

The final output from this analysis is a two-dimensional spatial map of current source densities aligned with the electrode layout in distinct hippocampal layers, providing a detailed visualization of synaptic activity. This enhanced mapping capability of kCSD offers greater resolution and sensitivity in detecting and localizing subtle synaptic modifications than traditional potential-based analysis.^68^ The clarity and precision in mapping synaptic events post-tetanic stimulation highlight dynamic synaptic interactions and connectivity changes. Utilizing kCSD in conjunction with the EvoNES platform has markedly advanced our understanding of the intricate dynamics of synaptic responses, which is crucial for exploring the mechanisms underlying neural circuit functionality and synaptic plasticity.

### F. Immunofluorescence protocol

Fixed mouse brains were sectioned horizontally at 14 *μ*m and processed for immunofluorescence using established protocols.^70^ The cryosections were first rehydrated in 1× PBS and then permeabilized progressively with Triton X-100, decreasing concentrations from 0.3% to 0.1% in PBS (PBST). Antigen retrieval was performed by heating the sections in 10 mM citric acid (pH 6.0) at 95 °C for 10 min, followed by cooling for 20–30 min and extensive washing in 0.1% PBST at room temperature. The sections were blocked in 0.1% PBST containing 5% normal goat serum for 1 h at room temperature. Subsequently, they were incubated with primary antibodies diluted 1:1000 in the blocking solution overnight (14–16 h) at 4 °C in darkness. After several washes, the sections were incubated with secondary antibodies and diluted 1:1000 in blocking solution for 2 h at room temperature. Following additional washing steps in 0.1% PBST and then 1× PBS, the sections were stained with Hoechst (1:1000 dilution from a 10 mg/ml stock solution, Thermo Fisher) for 30 min in darkness, washed thoroughly in 1× PBS, mounted with Fluoromount-G (Invitrogen, Germany), and left to air-dry overnight in darkness. The preparations were then sealed with nail polish (Electron Microscopy Sciences, Germany) and examined under an LSM 980 Airyscan 2 microscope (Zeiss, Germany). For immunolabeling, the following primary antibodies were used: Tuj1 (rabbit, Synaptic Systems, 302302, 1:500), Map2 (guinea pig, Synaptic Systems, 188006, 1:250), and NeuN (chicken, Synaptic Systems, 266006, 1:200). All primary antibodies underwent antigen retrieval to ensure optimal staining.

### G. Statistical analysis

All statistical analyses were performed with Python and Originlab 2024. All data in this work were expressed as the mean ± standard error of the mean (SEM). All box charts are determined by the 25th–75th percentiles and the whiskers by the 5th–95th percentiles and lengths within the interquartile range (1.5 IQR). Also, the lines show the median and the squares for the mean values. Differences between groups were examined for statistical significance, where appropriate, using the Kolmogorov–Smirnov test, one-way analysis of variance (ANOVA), followed by Tukey's posthoc testing. P‐value < 0.05 is considered significant, and n.s. indicated non-significant.

## SUPPLEMENTARY MATERIAL

See the supplementary material for real-time pseudo-color map videos illustrating large-scale baseline and tetanic-evoked potentiation (LTP) states. Movie 1 shows evoked activity across the CA1–CA3 network following Schaffer collateral stimulation, while Movie 2 captures DG network dynamics following perforant path stimulation.

## Data Availability

The authors affirm that the data underpinning the findings of this study are included within the article and its supplementary material files. Nonetheless, a substantial volume of multidimensional raw data, generated at the BIONICS Lab DZNE Dresden, is not publicly accessible. These data can be obtained from the corresponding author upon reasonable request. Similarly, the code used for data analysis is not publicly available at present due to restrictions linked to a license agreement. However, it can be provided by the corresponding author upon reasonable request.
